# Growth differentiation factor 9 and bone morphogenetic protein 15 expression in previtellogenic oocytes and during early embryonic development of Yellow-tail Kingfish *Seriola lalandi*

**DOI:** 10.1186/0717-6287-47-60

**Published:** 2014-11-20

**Authors:** Jaime Palomino, Giannina Herrera, Phillip Dettleff, Víctor Martínez

**Affiliations:** FAVET-INBIOGEN, Faculty of Veterinary Sciences, University of Chile, Avda. Santa Rosa 11735, La Pintana, Santiago Chile

**Keywords:** *gdf9*, *bmp15*, Embryo development, Oocyte maturation, Fish

## Abstract

**Background:**

During fish oocyte maturation, specific molecules are expressed and accumulated within oocyte until fertilization and embryo development. Special attention have been paid in members of the transforming growth factor (TGF-β) superfamily; growth differentiation factor 9 (GDF9/*gdf9*) and bone morphogenetic protein 15 (BMP15/*bmp15*), which exert regulatory functions during oocyte maturation and follicle development. However, little attention has been paid to the involvement of these molecules during embryogenesis considering its importance for the formation of a good quality egg and subsequent embryo survival. The purpose of this study was to analyze the expression of *gdf9* and *bmp15* in previtellogenic oocytes and during early embryonic development in *Seriola lalandi*, a pelagic fish with increasing prospect for its aquaculture development, which however, show high mortality at embryo and larval stages.

**Results:**

Through RT-qPCR it was found that *gdf9* expression was higher in previtellogenic oocytes decreasing after ovulation. This expression profile agrees with its participation in early stages of the follicular development. The transcripts for *bmp15* also showed the highest levels in previtellogenic oocytes, however this expression was lower than obtained with *gdf9*. Conversely, in recently spawned oocytes mRNA *bmp15* levels were highest than observed to *gdf9.* This, is consequent with the main role proposed for this growth factor at the final fish oocyte maturation: avoid the ovulation of an immature oocyte. During embryo development, low levels of mRNA were detected to *gdf9*, with an increase in 48 H post-fertilization embryos. The *bmp15* expression did not change throughout development and was higher than *gdf9* at 16 cells, blastula and appearance embryos stages.

**Conclusions:**

Both (*gdf9* and *bmp15*) expression profiles in previtellogenic oocytes and newly spawned eggs are consistent with the described functions for these growth factors in vertebrate ovarian physiology in early and late stages of the follicular development. So, these genes could be considered as quality biomarkers at these stages. However, further studies of these proteins throughout folliculogenesis, are necessaries to fully understand their functions during the oocyte formation. In addition, the persistent expression of these growth factors during development, allows us to speculate possible roles in embryonic processes, which must also be addressed.

## Background

In recent years, evidence regarding to the active role of the oocyte in its maturation during folliculogenesis and the effect on embryonic development, has been accumulated [1, 2]. This role is performed through molecules secreted by the oocyte which could regulate the folliculogenesis and steroidogenesis in both granulose and theca cells [3]. In mammals, some of the key molecules involved in these processes are the members of the transforming growth factor β super family (TGF-β), GDF9 and BMP15 [4, 5]. These growth factors stimulate the proliferation of granulose cells, but also suppress follicle stimulating hormone (FSH)-induced granulose cell differentiation [6, 7]. The effect of GDF9 on FSH action has been presumed by its ability to inhibit FSH-dependent luteinizing hormone (LH) receptor expression, cAMP production, and steroids synthesis [6, 8, 9]. Further, it is appear that GDF9 promotes follicular survival by suppressing granulose cell apoptosis and follicular atresia [10]. Similar to GDF9, BMP15 is an inhibitor of FSH-induced progesterone synthesis and *in vitro* studies have demonstrated that this effect is mediated through the suppression of FSH receptor gene expression [11]. BMP15 is also involved in the *cumulus* expansion by stimulating the epidermal growth factor (EGF)-like growth factor expression, which is crucial for *cumulus* cells to be capable of responding to LH-induced granulose cell signal [12]. It has been purposed that BMP-5 participate in the regulation of *cumulus* cell apoptosis, but the molecular mechanism it is still unknown [13].

Information about GDF9 and BMP15 in non-mammalian vertebrates is limited. However in order to understand the functional roles of these growth factors in those groups of animals, significant contributions principally related with its mRNA expression profiles have been made. In fishes, both *gdf9* and *bmp15* genes have been studied in zebrafish [14–16], carp [17, 18], eels [19, 20], European sea bass [21] and rainbow trout [22]. In general, it has been described a decreasing in *gdf9* expression during follicular development among fish species. In zebrafish and gibel carp, the highest *gdf9* expression levels has been observed in ovaries composing by oogonias and cells in transition to primary oocytes [16, 17]. By the contrary, in European sea bass, eels, rainbow trout and wuchang bream, the highest levels of *gdf9* mRNA have been revealed at the previtellogenic stage [18–22].

As well as *gdf9*, *bmp15* shows specie-specific patterns in both expression profiles and function during fish folliculogenesis. In zebrafish, important roles throughout follicle maturation by inhibiting premature follicles development and thus avoiding the ovulation of an immature oocyte have been suggested for BMP15 [15, 16, 23]. *In vitro* studies using zebrafish follicles, have revealed that incubation with human recombinant BMP15 or over expression in oocytes, resulted in an inhibition of oocyte maturation induced by both gonadotropins and maturation inducing hormone (MIH) [14, 15]. Therefore, these findings suggest that BMP15 modulates the follicular growth and prevents premature oocyte maturation in zebrafish, in part, by suppressing the sensitivity of follicles to MIH [23]. Furthermore, similar expression profile compared to *gdf9* was observed during fish follicular development of European sea bass, suggesting that these factors might act cooperatively in their ovarian functions [21].

These evidences suggest that as well as in mammals, the fish oocyte can function as a cellular signaling center during folliculogenesis, executing their functions through the production of growth factors which are maintained into the zygote after fertilization. These molecules are considered essential for a good quality egg, with a direct effect on embryonic and larval survival [16, 18, 24, 25]. Nevertheless, the involvement of these molecules in ovulated oocytes and during embryonic development has had little attention. Some studies performed in mice have correlated the presence of these proteins in the environment of oocyte maturation with the embryo viability [26, 27]. In this way, higher rates of development and percentage of hatching blastocyst resulted when GDF9 exogenous was added to culture medium during *in vitro* oocyte maturation [26]. In addition, high BMP15 level in follicular fluid was associated with high quality in the ovulated oocyte and its subsequent embryonic development [27]. Previously, it had been described that in mice, *gdf9* mRNA disappeared around 1.5 days after fertilization but the explanation was not discussed [28]. Unlike its mammalian ortholog, embryonic expression of amphibian BMP15 expends beyond early cleavage stages and is maintained throughout embryogenesis. In addition, it has been demonstrated that BMP15 function is both necessary and sufficient to the specification of head and trunk structures [29]. Expression of these growth factors during fish development has been studied in two species; wuchang bream *Megalobrama amblycephala* and zebrafish *Danio rerio*[16, 18]. In these species, the temporal expression pattern of *gdf9* showed highest level in zygote declining sharply near six hours post fertilization. However, although at low levels, this protein was detectable through advanced development stages in both species, which could indicate that it may be important to fish early embryonic development. Considering that in teleost the early embryonic development occurs principally inside the eggs with scarce exchange with the environment, the molecules involved in the oocyte maturation, such as growth factors GDF9 and BMP15, are very important in order to obtain a fully mature oocyte which should be capable of carry out the development after fertilization. Therefore, the study of these molecules in species with high mortality during embryo and larval stages is especially valuable.

The yellow-tail kingfish *Seriola lalandi* is a marine pelagic fish with a circumglobal distribution [30]. This species has been considered essential to diversify the Chilean aquaculture due to its rapid growth rate, good flesh quality and an increasing worldwide demand. However, the full production cycle of this species has been difficult to achieve due to high mortality during embryonic and larval stages [30, 31]. Previous studies about the reproductive physiology of wild caught individuals established that males and females reach 50% maturity at a fork length of 812 and 944 mm, respectively. The spawn occur naturally during the austral spring and summer when the seawater temperature was above 17°C and individuals spawn repeatedly during this time [31]. It has been observed a courtship behavior which consisted of a high-speed pursuit punctuated by stalling, nipping and touching between one male and female [30]. There is no scientific evidence regarding disorders during embryogenesis in *S. lalandi* to explain this low survival, and there are not antecedents regarding to aspects involved in oogenesis and early development in this species. Studies performed in marine fish have proposed that low buoyancy of eggs and early embryos [32], skeletal malformations [33], arrested eye migration [34] and locking of the jaw cartilage [35] could be the result of inappropriate oocyte maturation. Thereby, to elucidate molecular aspects involved in oocyte maturation and early embryogenesis, appear to be essential in order to understand and eventually improve the reproduction in this species. Therefore, the objective of this work was to study the expression of growth factors *gdf9* and *bmp15* in previtellogenic oocytes and during early embryonic development in *S. lalandi*.

## Results

### RT-qPCR conditions

The RNA quality was evaluated according to denaturing agarose electrophoresis gels and we only worked with those samples where it was possible clearly to distinguish both bands that constituted the total RNA (18 and 28 sub-unities) without an important smears (data not shown). For each primer pairs, non-specific amplification was evaluated using the shape of the melting curves (Tm), where it was possible to observe a single clear, with a Tm similar to what is expected based on the amplicon sequence. Furthermore, for all primers pairs used in this work, the optimum combination between forward and reverse primers concentration was determined and the selection criteria used was the lower Ct value and smaller standard deviation between replicates of the amplification curves in experiments with the same cDNA template concentration. Finally, it was crucial to validate the qPCR results with efficiencies near to 2 (100%). All reactions displaying similar efficiencies are presented in Table 1.

**Table 1:** **PCR characteristic of primers for constitutive and growth factors genes evaluated in the present study**

Gene	Primer sequences (5′-3′)	Tm (°C)	Efficency	Amplicon (bp)
*β-actin*	F: AGGGAAATCGTGCGTGACAT	57	2,04	191
	R: GCTGAAGTTGTTGGGCGTTT			
*tubulin*	F: TCATCAAGATTATCAGGAGGCG	55	1,98	113
	R: GGAAGCATACACCATGTAGAGG			
*gdf9*	F: ACGAATGCATGTGCCTTGTA	55	2,01	186
	R: GCTTCTCGTAAATGATGTTCTG			
*bmp15*	F: GGCAAAGAGAGCCTGGTCACC	59	1,99	218
	R: GAAGGTGGAGTACAGGGAGCT			

### Growth factors expression during embryonic development

**Figure 1:**
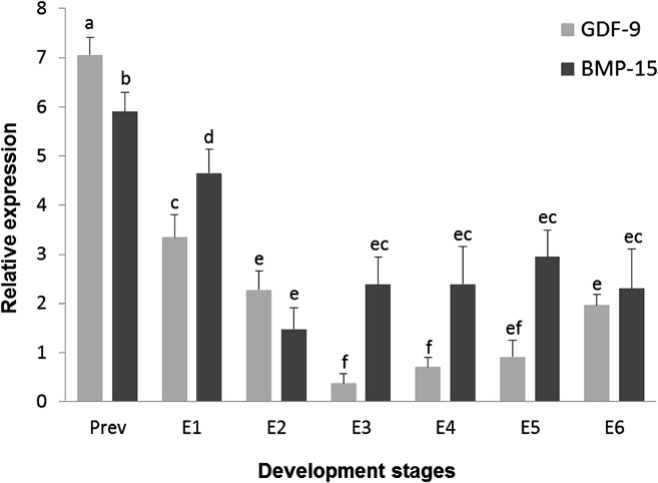
**Relative mRNA levels of gdf9 and bmp15 throughout development in** ***S. lalandi*** **.** The bars represent the means ± standard deviations of growth factors relative expression in previtellogenic oocytes (Prev) and during different developmental stages in *S. lalandi* (E1: newly spawned eggs; E2: 4 cells, E3: 16 cells, E4: blastula, E5: appearance embryo and E6: advanced embryo). Same letters indicate no significant differences (p >0,05).

RT-qPCR was performed to assess the relative expression levels of both *gdf9* and *bmp15* in previtellogenic oocytes and throughout the *S. lalandi* embryonic development. The higher expression levels of *gdf9* transcripts were observed in previtellogenic oocytes and a significant decrease in the expression of this growth factor was observed after ovulation (Figure 1). Further, as embryonic development progresses, the *gdf9* mRNA expression declined dramatically at 16 cells stage (E3) maintaining low levels even after gastrulation process (E5) (Figure 1). Interestingly, a significant increase in the *gdf9* expression was observed after 48 hours post fertilization (E6), in spite of this expression level was lower than observed in previtellogenic and recently spawned oocytes (Figure 1). Similar to *gdf9*, transcripts to *bmp15* showed the highest levels in previtellogenic oocytes, but its expression was lower than *gdf9*. After spawn and fertilization, was observed a significant decrease in the *bmp15* expression, but at this stage (E1), highest mRNA levels than *gdf9* was found. In 4 cell embryos (E2), a large decrease in the *bmp15* expression was observed and no changes in these transcripts levels were observed through development (Figure 1). From 16 cells stage (E3) until embryos 24 hour post fertilization (E5), *bmp15* expression was higher than *gdf9*. Finally, in 48 hour embryos (E6) no differences in the expression of these growth factors were detected (Figure 1).

## Discussion

Although mRNA levels to *gdf9* and *bmp15* during follicular development have been analyzed in several teleost species, there is little knowledge regarding the expression of these growth factors after fertilization and their possible biological significance in both embryonic and larval survival. Therefore, given the fundamental role of these TGF-β members in the production of a good-quality oocyte, this study has attempted to give preliminary evidence regarding the expression of these growth factors in previtellogenic oocytes and during early embryonic development of yellow tail Kingfish *S. lalandi*.

The expression level of *gdf9* was clearly highest in previtellogenic oocytes compared with its expression during embryo development. These results agree with studies in rainbow trout, eels and European sea bass, where the highest level of *gdf9* expression at the previtellogenic stage has been revealed, with an important decline along the vitellogenic process [19–22]. Although GDF9 functions in *S. lalandi* are currently unknown, its high expression in previtellogenic oocytes would propose a role of this growth factor during early ovarian development with potential participation in the follicular recruitment or thereafter during early stages of the vitellogenic process. More accurate conclusions about the functions of this growth factor in *S. lalandi* follicular growth must be elucidated by studying its expression at different stages of ovarian development. However, based on our results and comparing its expression levels, between previtellogenic occytes and recently spawned eggs (E1), we can infer a decrease in the expression of this growth factor during vitellogenesis, which is consistent with the observed in rice field eel [20], zebrafish [16] and European sea bass [21]. Similarly, the *bmp15* mRNA levels were significantly lower in ovulated oocytes in comparison with previtellogenic oocytes. These results would indicate a decrease in the *bmp15* expression during follicular development and more specifically during vitellogenesis and maturation. This interpretation is consistent with studies in gibel carp [36] and European sea bass [21], where a decrease in the *bmp15* expression was observed through vitellogenic process. On the contrary, *bmp15* in zebrafish, was expressed consistently in follicles of different developmental stages and no significant differences were observed in their expression levels [14].

The expression patterns for *gdf9* and *bmp15* in previtellogenic oocytes confirm the involvement of these proteins in the early stages of follicular development. However, the difference in the expression levels between these growth factors was an interesting result. In previtellogenic oocytes, the levels of *gdf9* mRNA were higher than those of *bmp15*, which can be explained by its critical role during early stages of follicular development, specifically during recruitment [16]. Furthermore, this observation supports the hypotheses for European sea bass where it has been suggested that *gdf9* expression appears not to be regulated by gonadotropins [37]. By contrast, spawned eggs showed higher levels of *bmp15* mRNA in comparison with *gdf9* expression, which agree with the principal role proposed for *bmp15* in zebrafish, in maintaining egg quality and preventing ovulation of an immature oocyte [23]. Major conclusions regarding the importance of these growth factors and their coordination in processes such as vitellogenesis and oocyte maturation should be obtained by studying their expression during different stages of follicular development.

On the other hand, during *S. lalandi* embryonic development time course, both *gdf9* and *bmp15* expression levels significantly decreased after two hours post fertilization (E2). Specifically, *gdf9* showed the lower expression value in 16 cell embryos (E3), after two and half hours post fertilization. This situation, agrees with that observed in wuchang bream [18], but differs which occurs in zebrafish, where a high *gdf9* expression is maintained until 6 hours post fertilization, at the blastocyst stage, decreasing with the advanced development [16]. We believe that the different lengths of the embryonic processes among fish species could be the explanation for the differences in the *gdf9* expression. Therefore, despite there is no evidence to support the biological roles of this growth factor at these development stages, its decreased level of mRNA during the early embryonic development suggest that its gene expression may be affected by the gradual transition period from oocyte-derivate mRNA and proteins to full embryonic transcriptions [38]. In mammalian embryo development, *gdf9* has been identified as a part of maternal mRNA to be used during early embryogenesis [25], so a similar situation may also be occurring in *Seriola*, as well as in other fish species. In the other hand, an interesting and significantly increase in the *gdf9* expression was observed in 48-hour post fertilization embryos (E6). A different situation was observed in zebrafish, where *gdf9* mRNA virtually disappeared from the gastrula stage, about 10 hours after fertilization [16]. However, our result would agree with those reported in wuchang bream, where despite the *gdf9* mRNA is maintained at a low level, a significant increase was detected in 62 hours embryos compared with those of 50 hours post fertilization [18]. Therefore, the persistent expression of *gdf9* in early *S. lalandi* embryos would implicate functions of this molecule in early embryogenesis as well as it has been proposed to others growth factors such as GDF1 [39] and GDF5 [40], which have been implicated in the left right patterning and skeletal joint formation, respectively.

The *bmp15* level expression decrease significantly after two hours post fertilization (E2) and, unlike *gdf9*, its mRNA levels did not vary as the embryo development was progressing. Nevertheless, between 16-cell (E3) and appearance embryos (E5) stages, higher expression levels for *bmp15* in comparison to *gdf9,* were observed. The involvement of *bmp15* in teleost embryogenesis has not been yet described. However, changes in its structure that includes a serine domain, similar to that found in yolk proteins linked to calcium phosphate transport during the embryo bone formation, has been mentioned to speculate its role in this process during fish embryogenesis [21].

## Conclusions

The expression of *gdf9* and *bmp15* in previtellogenic oocytes and during early embryonic development in *S. lalandi*, has been confirmed. The consistency between their expression profiles in previtellogenic oocytes and newly spawned eggs, with the proposed functions in mammals and other fish species, would allow proposing the possibility of use these genes as potential quality biomarkers at these stages. However, the biological significance of their expression during embryonic development should be more depth studied.

## Methods

### Samples collection

All animals used in the present study as well as the experimental procedures were treated according the Chilean Bioethics Committee of the National Commission for Scientific and Technological Research. The samples were provided by Acuinor S. A., a commercial company that has successfully developed the complete production in captivity in a hatchery production center located in Caldera, Atacama Region, Chile. The broodstock comprise 40 individuals maintained in two indoor tanks with 2.5 m depth and 20,000 Lts, in a sex proportion of 1:1. Animals were subjected to different photoperiods, temperatures between 18 to 20°C and daily feeding *at libitum*, in order to assure the availability of eggs around the whole cycle. The spawned eggs were channeled from a skimmer on the surface of each tank into a separate egg collector. Three batches of each tank were observed from spawning and samples of approximately 100 individuals were taken at different development stages as described by Moran et al. [30]. The selected development stages were: newly spawned eggs, 4 cell, 16 cell, blastula, appearance embryo and advanced embryo, which were identified as E1, E2, E3, E4, E5 and E6 respectively. In addition, ovary samples obtained by cannulation of the gonophore of three anesthetized adult female individuals were stirred in a petri dish with 3 mL of micro filtered sea water. Then approximately 200 previtellogenic oocytes that ranged between 30 and 50 μm were taken with a fine bore pipette. All samples were maintained in RNA*later*® Solution (Ambion®) and processed to RNA extraction.

### RNA extraction and cDNA synthesis

Total RNA was extracted using the column affinity Purification Kit GeneJET™ RNA (Fermentas Life Sciences) following the manufacturer’s instructions. The RNA concentration was determined using a Qubit® Fluorometer (Invitrogen™) with the quantification kit Qubit® RNA Assay, (Molecular Probes® Invitrogen™). DNA contamination was removed by Dnase I treatment and the total RNA quality was assessed by 1% ethidium bromide agarose gels electrophoresis under denaturing conditions. The RNA samples were stored at -80°C until use. The samples were processed for reverse transcription (RT) using the enzyme conjugate SuperScript™ First-Strand Synthesis System (Invitrogen™). In order to corroborate the absence of contaminating DNA, RT negative control was used for each sample. The complementary DNA (cDNA) concentration was determined using the Quantification Kit ssDNA Qubit® Assay (Invitrogen™ Molecular Probes®). cDNA samples were stored at -20°C until use in qPCR protocols as described below.

### Primer design

Primers for growth factors and two constitutive genes were designed using information obtained from Genbank EST sequences in different fish species. Recently we determined that both *β-actin* and *tubulin* are suitable pair of constitutive genes for data normalization in RT-qPCR studies during embryonic development of *S. lalandi* (data no published). Primers to constitutive genes were generated from *Seriola quinqueradiata* EST sequences (Accession numbers (AN): AB179839.1 and AB461862.1 for *β-actin* and *tubulin*, respectively). To *gdf9* primers were designed by sequence alignment from four fish species (*Dicentrarchus labrax*, AN: AM933667.1; *Oncorhynchus mykiss*, AN: EU723245.1; *Carassius gibelio*, AN: HQ454282.1 and *Danio rerio*, AN: AY833104.1). Primers to *bmp15* were designed based on three fish species sequences (*Carassius gibelio*, AN: HQ454283.1; *Cyprinus carpio,* AN: JN002405.1 and *Dicentrarchus labrax,* AN*:* AM933668.1). The alignment was performed using the ClustalX program and the primers were searched in about 200 bp highly conserved regions. All primers were designed with Primer 3Plus considering a theoretical optimal annealing temperatures about 60°C, with differences between the forward and reverse primers not greater than 5°C, guanine and cytosine percentages between 30 and 60%, primers lengths 18 to 20 bp and free of dimmers and hairpins. In addition, all primers were tested by NET primer (http://www.PremierBiosoft.com/netprimer). All the primers are showed in Table 1.

### Quantitative real-time PCR (qPCR)

Gene amplifications by qPCR were performed with an Illumina® Eco Real Time PCR System Model EC-100-1001. Each 12.5 μL reaction contained the following: 6.25 μL of Maxima SYBR Green/Fluorescein qPCR Master Mix (2X), a necessary volume to 0.2-0.6 μM of each primer, a volume with 10 ng of cDNA and the volume difference was complete with DEPC treated water. Plates were sealed with adhesive optical film and the following PCR conditions were performed: after an initial denaturation step during 15 min at 95°C, 40 amplification cycles were performed according to the following thermo cycling profile: denaturation for 15 s at 94°C, annealing for 30 s at a temperature according to specific Tm of each primers pair and extension for 30 s at 72°C. A dissociation protocol with a gradient from 60 to 95°C was used to investigate the specificity of the qPCR reaction and the presence of primer dimers. Control samples without reverse transcriptase and without the template, were included in each plate. Prior to expression analysis, we standardized and optimized the qPCR conditions for all primers using cDNA obtained from ovarian samples, assessing the optimal concentration for each primer pair and their efficiency in the PCR. The efficiency (E) was evaluated with six-point standard curves of a six-fold dilution series (1:1-1250). The slope of the standard curve represents the amplification efficiency (E) [41], through the following formula E =10^(‒1/slope)^. We used only primers pairs with efficiencies approaching 2 (which indicate 100%) and over 0.989 of R^2^ value [42].

### Data analysis

After standardization of PCR conditions, we evaluated the relative expression of *gdf9* and *bmp15* genes in previtellogenic oocytes and during different embryo development stages in *S. lalandi*. Three biological replicates that represent three different spawning events were analyzed, which were evaluated in three technical replicates. In each experiment, gene expression levels were recorded as Ct values that corresponded to the number of cycles where the fluorescence signal can be detected above a threshold value. The Cts averages for each biological replicate were calculated and transformed into relative values denominated *Quantity* (Q) through ΔΔCt method [43]. Then, the relative quantification in the expression of *gdf9* and *bmp15* for each developmental stages was estimated as the quotient between Q value of the target gene and a normalization factor (NF), which was calculated based on the geometric mean of reference genes Q values [43]. Relative expression was expressed as the mean ± standard deviation and the data were analyzed by ANOVA using InfoStat Professional Program, Version 2004; National University of Córdoba, Argentina. Significant differences among means were evaluated using Tukey test and values were considered significantly different when P <0.05.
